# Fine mapping of a leaf flattening gene *Bralcm* through BSR-Seq in Chinese cabbage (*Brassica rapa* L. ssp. *pekinensis*)

**DOI:** 10.1038/s41598-020-70975-2

**Published:** 2020-08-18

**Authors:** Meidi Zhang, Shengnan Huang, Yue Gao, Wei Fu, Gaoyang Qu, Yonghui Zhao, Fengyan Shi, Zhiyong Liu, Hui Feng

**Affiliations:** Department of Horticulture, Shenyang Agricultural University, Shenyang, China

**Keywords:** Developmental biology, Genetics, Plant sciences

## Abstract

Leaf flattening influences plant photosynthesis, thereby affecting product yield and quality. Here, we obtained a stably inherited leaf crinkled mutant (*lcm*), derived from the Chinese cabbage doubled haploid (DH) ‘FT’ line using EMS mutagenesis combined with isolated microspore culture. The crinkled phenotype was controlled by a single recessive nuclear gene, namely *Bralcm*, which was preliminarily mapped to chromosome A01 by bulked segregant analysis RNA-seq, and further between markers SSRS-1 and IndelD-20 using 1,575 recessive homozygous individuals in F_2_ population by a map-based cloning method. The target region physical distance was 126.69 kb, containing 23 genes; the marker SSRMG-4 co-segregated with the crinkled trait. Further, we found SSRMG-4 to be located on *BraA01g007510.3C*, a homolog of *AHA2*, which encodes H^+^-ATPase2, an essential enzyme in plant growth and development. Sequence analysis indicated a C to T transition in exon 7 of *BraA01g007510.3C,* resulting in a Thr (ACT) to Ile (ATT) amino acid change. Genotyping revealed that the leaf crinkled phenotype fully co-segregated with this SNP within the recombinants. qRT-PCR demonstrated that *BraA01g007510.3C* expression in *lcm* mutant leaves was dramatically higher than that in wild-type ‘FT’. Thus, *BraA01g007510.3C* is a strong candidate gene for *Bralcm*, and *AHA2* is possibly associated with leaf flattening in Chinese cabbage.

## Introduction

The leaf is a vital vegetative organ that provides energy for plant growth and development through photosynthesis, respiration, and by storing nutrients^1,2^. Leaf morphology is an important feature of plant architecture that significantly affects yield performance^3–5^. The control of leaf morphology is a complex physiological and biochemical process, regulated by the expression of many functional genes and transcriptional regulatory factors^6^.

Numerous genes involved in leaf morphology have recently been identified. *YABBY* genes were first identified by Siegfried et al.^7^. *YABBY* members are involved in the promotion of abaxial fate in Arabidopsis. *WOX* genes have several members including *WOX1* and *WOX3/PR*S, which play a major role in leaf development^8^. HD-ZIP III family members have an important role in leaf adaxial fate determination^9–11^. The *LATERAL ORGAN BOUNDARIES DOMAIN* (*LBD*) family genes participate in the morphogenesis of plant lateral organs^12,13^, such as *AtLBD6*, which control cell proliferation on the adaxial surfaces of leaves, leading to the development of flat leaves with bilateral symmetry^14^. H^+^-ATPase 2 (AHA2) is an essential enzyme for plant growth, which is involved in the transmembrane transport, the elongation and growth of cells, the opening and closing of stomata, the response of plants to environmental stress and other physiological processes. It is auto-regulatory and affects gene expression through a variety of regulatory systems to adapt to the changes in the environment during the development process of plants^15^. Leaves with different morphologies have different light energy utilization efficiencies, which may result in yield differences. Therefore, it is important to study the genes which affect the development of leaf morphology.

Appropriate leaf rolling is an important element in the ideotype model of plant breeding, as it can increase yield by improving photosynthetic efficiency and reducing leaf transpiration under drought stress. However, excessive leaf rolling can lead to growth retardation and yield reduction^16^. In plants, many mutants associated with leaf flatness have been found. Fang et al.^17^ obtained the rumpled and twisted leaf 1 (*rtl1*) mutant using the ethyl methanesulfonate (EMS) treatment in rice. The *rtl1* locus with the phenotype of rumpled and twisted leaf was mapped to chromosome 4 using Bulked Segregant Analysis (BSA) sequencing, with a genetic distance of 1.47 cM. In upland cotton, a wrinkled leaf mutant (*wr3*) was identified and found to be controlled by a recessive gene, and the *wr3* gene was mapped on chromosome 21 using BSA sequencing^18^. A dwarf cucumber mutant (*scp-2*) with dark green wrinkled leaves was obtained and the *scp-2* gene was mapped to a 30.75 kb region on chromosome 3 using map-based cloning; *CsDET2*, which is involved in brassinosteroid (BR) synthesis, was identified as a candidate gene^19^. A mutation in the crinkled leaves 8 gene (*cls8*) was acquired by EMS treatment in *Arabidopsis thaliana*, and the *cls8* gene was mapped to a region of 39.2 kb on chromosome 2. The candidate gene identified was *At2g21790*, which encodes the large subunit of ribonucleotide reductase (RNR1)^20–22^. Hsieh et al.^23^ found an *Arabidopsis SLOW GROWTH3* (*slo3*) mutant, which had an obvious phenotype with severe growth retardation and curled or crinkled rosette leaves, which may be due to uneven growth of the leaf surface. The candidate gene identified was *At3g61360*, which encodes a pentatricopeptide repeat (PPR) protein. These mutants are the ideal materials for studying the regulatory mechanism of maintaining leaf flattening.

Chinese cabbage is an economically and nutritionally important vegetable crop, and is widely cultivated in Eastern Asia^24^. The leaf is the main product organ of Chinese cabbage. The size and shape of the leaf directly affects its attraction to consumers. In this study, a stably inherited leaf crinkled mutant (*lcm*) was derived in Chinese cabbage by EMS treatment combined with isolated microspore culture. We performed phenotypic characterization, genetic analysis, and fine mapping of *lcm*, and a high-resolution genetic map was constructed using simple sequence repeats (SSR) and insertion/deletion (indel) molecular markers. In the target region, the *BraA01g007510.3C* was the most likely candidate gene. This study lays the foundation for elucidating the molecular mechanism of leaf flattening in Chinese cabbage.

## Results

### Morphological characterization of *lcm* mutant

**Figure 1:**
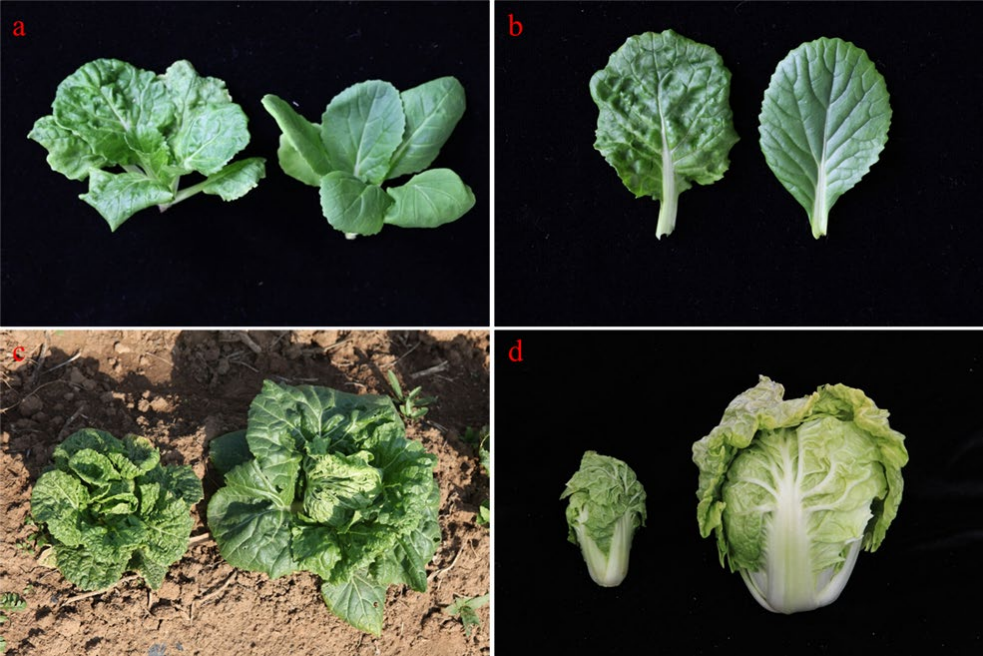
Phenotypic characterization of the wild-type ‘FT’ and the *lcm* mutant. (**a**) *lcm* (left) and wild-type ‘FT’ (right) at seedling stage, (**b**) the fifth true leaf of *lcm* (left) and wild-type ‘FT’ (right) with 35 days, (**c**) *lcm* (left) and wild-type ‘FT’ (right) at heading stage, (**d**) leafy heads of the *lcm* (left) and wild-type ‘FT’ (right).

The leaves of the *lcm* mutant were crinkled and developed slowly at all stage compared with ‘FT’ (Fig. 1a–c). However, at the heading stage, the crinkled phenotype of leaves in *lcm* mutant was the most obvious and the leafy head was remarkably smaller than ‘FT’ (Fig. 1c,d, Table S1). The flow cytometry results indicated that the *lcm* mutant was diploid (Fig. S1). The growth curve showed that the difference in leaf length, leaf width, and plant width were significant at 9–15 days after the third true leaf appeared (Fig. S2a–c), and the dry weight and fresh weight were all significantly reduced in *lcm* compared to the wild-type ‘FT’ (Fig. S2d,e).

**Figure 2:**
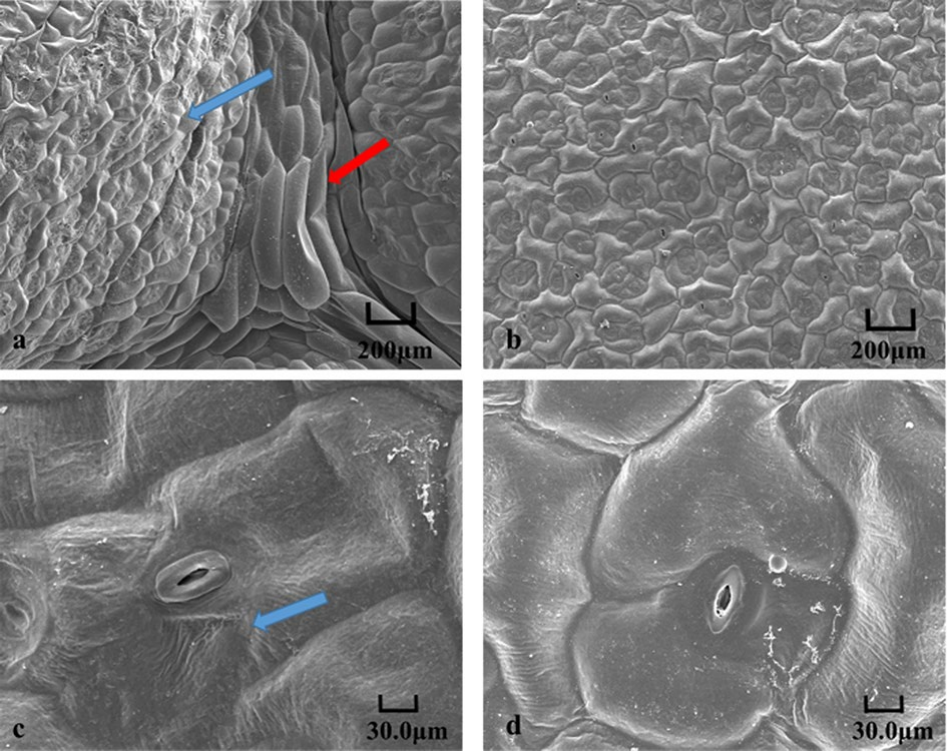
Observation of the leaf epidermal cells by SEM. (**a**) *lcm* mutant leaf, (**b**) wild-type leaf, (**c**) enlarged view of *lcm* crinkled cells, (**d**) enlarged view of wild-type cells. Arrows indicated the different parts.

**Figure 3:**
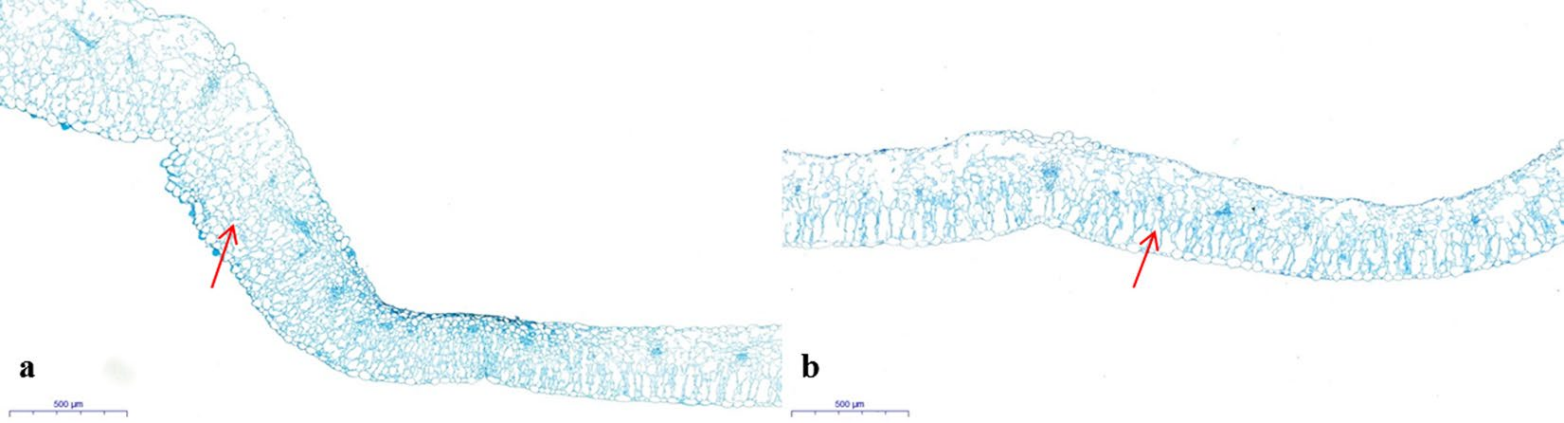
Observation of the leaf cross section by paraffin section. (**a**) *lcm* mutant leaf, (**b**) wild-type leaf. Arrows indicated the palisade cell.

The scanning electron microscopy (SEM) (Hitachi, Japan) result revealed that at the site of the leaf folds in *lcm* mutant, the cells were squashed into longer and thinner than in ‘FT’ (the red arrow) (Fig. 2a,b), and in the leaf crinkled part of *lcm* mutant, the cells also became crinkled (the blue arrow) (Fig. 2c,d). The paraffin section showed that the number of palisade cell was increased in the crinkled part of the mutant leaf than ‘FT’ (Fig. 3).

### Genetic analysis

The reciprocal cross F_1_ plants exhibited the same phenotype as ‘FT’, suggesting that the phenotype of *lcm* mutant is controlled by a recessive gene. Among 137 F_2_ individuals, 34 plants showed the mutant phenotype, whereas the others were the wild-type phenotype. The segregation ratio of the *lcm* phenotype corresponded to the expected ratio of 3:1 for ‘FT’ and *lcm* mutant (χ^2^ < χ_0.05_^2^ = 3.84) (Table 1). A total of 24 *lcm* phenotype individuals were shown in 50 BC_1_ individuals, the others were the wild-type. The segregation ratio was corresponded to the expected ratio of 1:1 between *lcm* mutant and ‘FT’ (χ^2^ < χ_0.05_^2^ = 3.84) (Table 1). These results indicated that the mutant character was controlled by a single recessive nuclear gene, namely *Bralcm*.

**Table 1:** Genetic segregation of the *lcm* in crosses between wild-type line ‘FT’ and *lcm* in Chinese cabbage.

Generation	Total	‘FT’	*lcm*	Segregation ratio	χ^2^
P_1_(‘FT’)	50	50	0		
P_2_(*lcm*)	48	0	48		
F_1_(P_1_ × P^2^)	53	53	0		
F_1_(P_2_ × P_1_)	49	49	0		
BC_1_(F_1_ × ‘FT’)	81	81	0		
BC_1_(F_1_ × *lcm* )	50	26	24	1.083:1	0.100
F_2_	137	103	34	3.029:1	0.079

### Preliminary mapping of *Bralcm* by bulked segregant RNA-seq (BSR-Seq)

*Bralcm* was preliminarily mapped using BSR-Seq. A total of 49,089,636 and 53,868,502 clean reads were obtained from mutation pool and normal pool, respectively, and the Q20 (those reads with an average quality score > 20) was > 93% and GC content was approximately 48%, suggesting that the sequencing was highly accurate (PRJNA565107) (Table S2). The clean reads were aligned to reference genome and a total of 38,792,091 and 42,976,949 single-nucleotide variants (SNVs) were obtained from the mutation and normal pools, respectively. Mutant loci with sequencing coverage depth greater than 3X were screened and the Euclidean distance^5^ (ED^5^) value of SNV was calculated. Based on the distribution of SNV, the linkage of ED^5^ value was plotted (Fig. S3). *Bralcm* was located on chromosome A01, using the top 1% of ED^5^ values as the correlation threshold, the target region was identified as chromosome A01 1016464–9172697 (Table S3).

**Figure 4:**
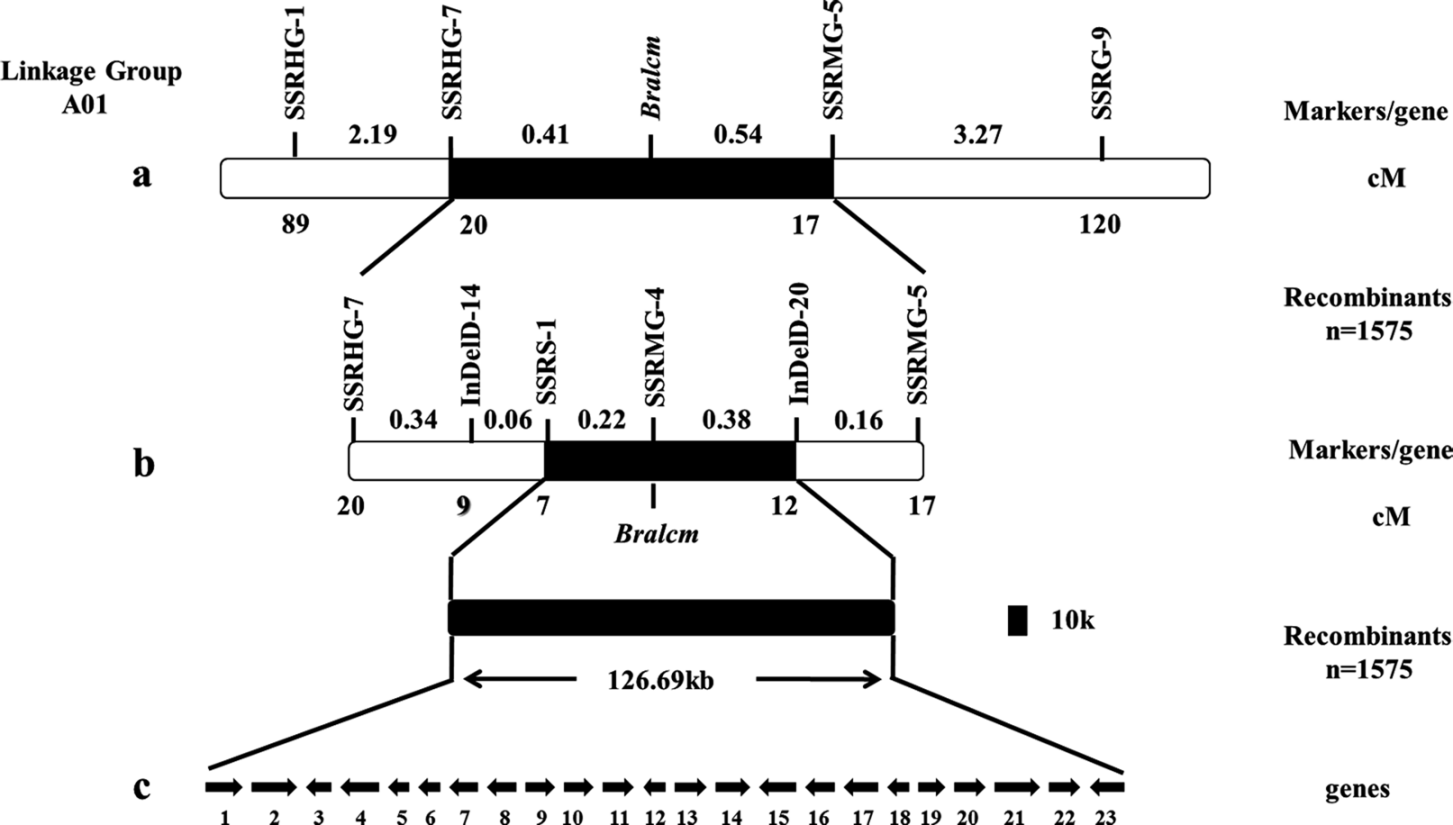
Genetic and physical maps of the *Bralcm* locus and candidate gene analysis. (**a**) Preliminary mapping of *Bralcm*. (**b**) Fine mapping of *Bralcm*. (**c**) Candidate gene analysis of *Bralcm*. Arrows indicate the direction of gene transcription. The numbers above the chromosome represent the genetic distance, and blow the chromosome represent the numbers of recombinants.

A total of 200 F_2_ recessive homozygous individuals with the mutant phenotype and by two parental lines (‘FT’ and *lcm*) were used to validate the result of BSR-Seq. The result showed that two SSR markers SSRHG-1 and SSRG-9 were in linkage with *Bralcm* (Table S4, Fig. S4), and *Bralcm* was mapped between markers SSRHG-1 and SSRG-9 on the chromosome A01 (Fig. 4a), with genetic distances of 2.6 cM and 3.81 cM, respectively.

### Fine mapping of *Bralcm*

New SSR and indel markers were designed between markers SSRHG-1 and SSRG-9. Six polymorphic markers SSRHG-7, IndelD-14, SSRS-1, IndelD-20, SSRMG-5, and SSRMG-4 were screened (Table S4). A total of 1,575 F_2_ plants with the mutant phenotype were used as the mapping population. The label of recombinants indicated that SSRHG-7, IndelD-14, SSRS-1were located on one side, and IndelD-20, SSRMG-5, and SSRMG-4 were located on the other side of *Bralcm* gene. *Bralcm* was mapped between SSRS-1 and IndelD-20. SSRS-1 had seven recombinants with *Bralcm*, the genetic distance was 0.22 cM, and twelve recombinants were different from SSRS-1 were detected between IndelD-20 and *Bralcm*, the genetic distance was 0.38 cM. The physical distance was approximately 126.69 kb (Fig. 4b). However, no recombinant was found in SSRMG-4, which is between SSRS-1 and IndelD-20 (Fig. 4b).

### Candidate gene analysis

**Figure 5:**
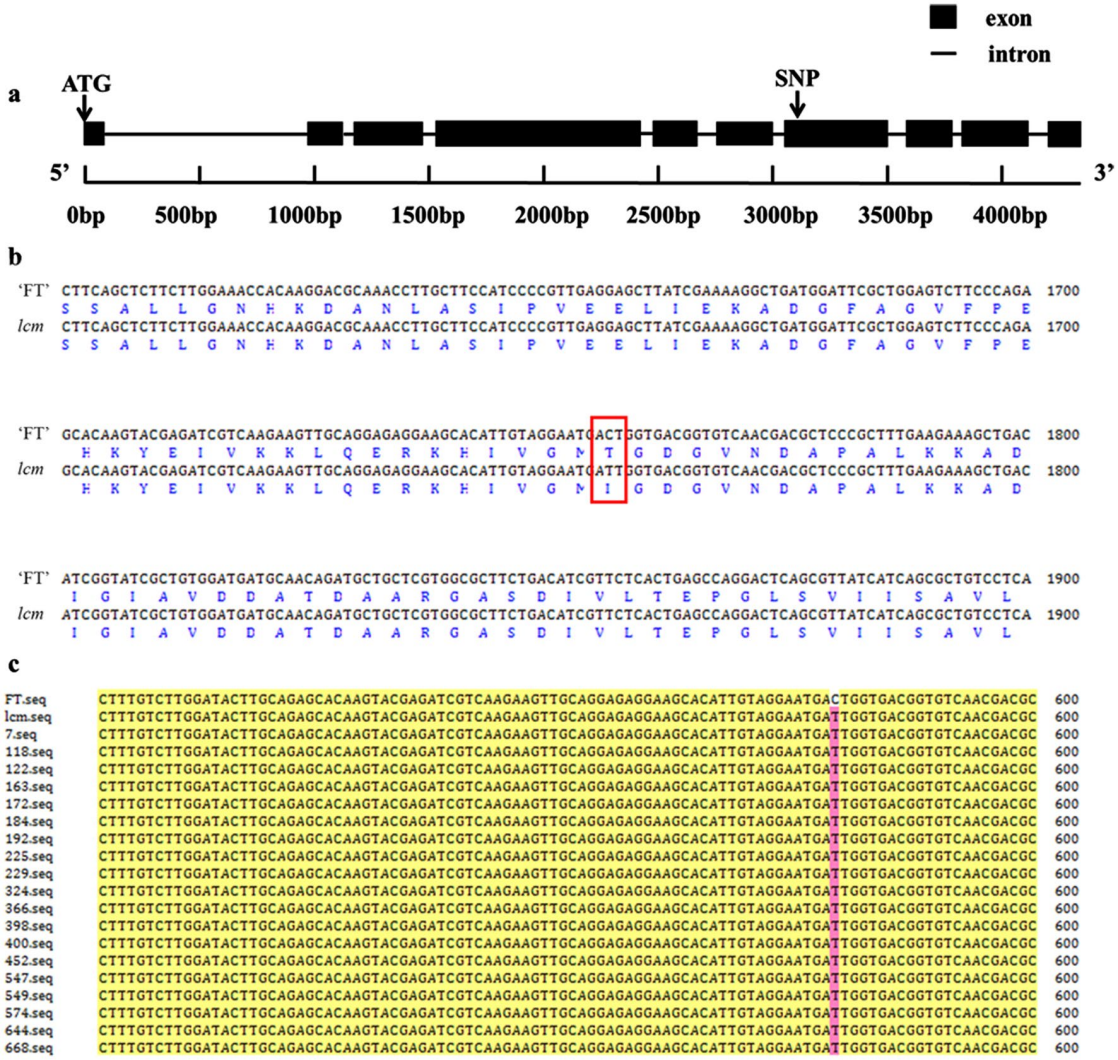
Gene structure and amino acid sequence alignment. (**a**) Gene structure of *BraA01g007510.3C* with the site of the non-synonymous SNP. (**b**) Alignment of the nucleotide and amino acid sequences of *BraA01g007510.3C* in the wild-type ‘FT’ and *lcm* mutant. The site created by the non-synonymous SNP is shown by an empty box. (**c**) Alignment of the nucleotide sequence of *BraA01g007510.3C* in nineteen F_2_ recombinants of the *lcm* mutant and wild-type ‘FT’. Nineteen F_2_ individuals were recombinants of the most closely linked markers SSRS-1 and InDelD-20.

According to the result of fine mapping and the Brassica database (https://brassicadb.org/brad/index.php), there were 23 genes in the target region (Fig. 4c, Table S5). Among them, the marker SSRMG-4 was co-segregation with the mutant trait and contained in *BraA01g007510.3C*, a homolog of AHA2 which encodes an H^+^-ATPase2 (AHA2) and is one of the vital enzymes in plant growth and development. Therefore, seven pairs of primers were designed along the full length of *BraA01g007510.3C* for cloning in ‘FT’ and *lcm* mutant (Table S4). Cloning and sequencing results showed one SNP present in the *lcm* mutant, a C to T missense transition, leading to an amino acid codon to change from Thr (ACT) to Ile (ATT) (Fig. 5a,b, Fig. S5).

### Co-segregation analysis

The nineteen recombinants were further cloned between the two closest markers, SSRS-1 and InDelD-20. As shown in Fig. 5c, the sequences of the nineteen recombinants were in line with *lcm*, verifying the co-segregation of this SNP and the leaf crinkled trait. Therefore the *BraA01g007510.3C* was identified as the strongest candidate gene for the *lcm* mutant.

### Expression of candidate gene *BraA01g007510.3C*

**Figure 6:**
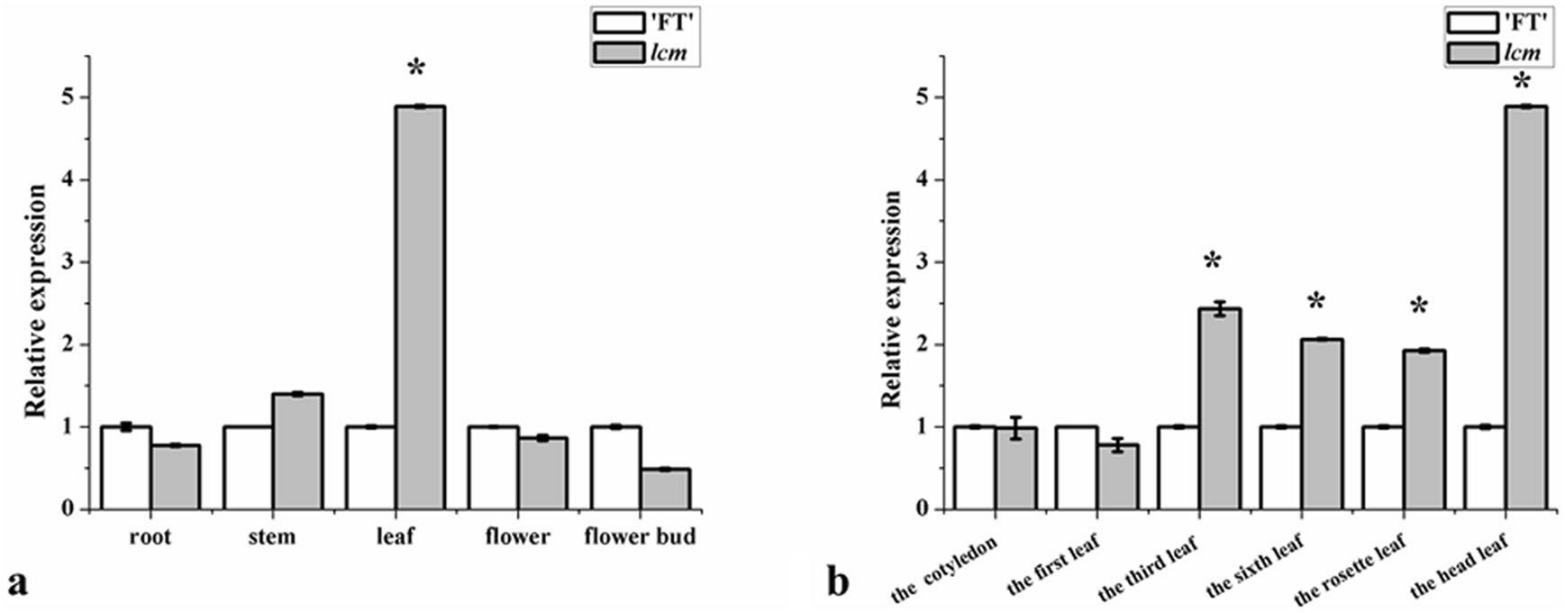
Expression analysis of *BraA01g007510.3C* by qRT-PCR. (**a**) Different organs (root, stem, leaf, flower, flower bud). (**b**) Different periods of leaf (cotyledon, the first true leaf, the third true leaf, the sixth true leaf, rosette leaf, head leaf). Asterisks indicate significant difference between the *lcm* and ‘FT’ (t test, P < 0.05).

Expression levels of *BraA01g007510.3C* was examined in the *lcm* mutant and ‘FT’ by quantitative reverse transcription PCR (qRT-PCR). The results showed that the expression of *BraA01g007510.3C* in leaves were dramatically higher in the *lcm* mutant than in ‘FT’ (Fig. 6a). To further analyze gene expression patterns, the expression of *BraA01g007510.3C* in leaves at different periods was analyzed in both the *lcm* mutant and ‘FT’. The qRT-PCR results showed that, compared to ‘FT’, the expression of *BraA01g007510.3C* in *lcm* mutant was up-regulated the third true leaf, in the sixth true leaf, rosette leaf and head leaf (Fig. 6b), and notably as the expression level of *lcm* mutant was highest in the head leaf and was consistent with the phenotype, the leaf was the most crinkled at heading stage.

### Enzyme activity and bioinformatics analysis of AHA2

**Figure 7:**
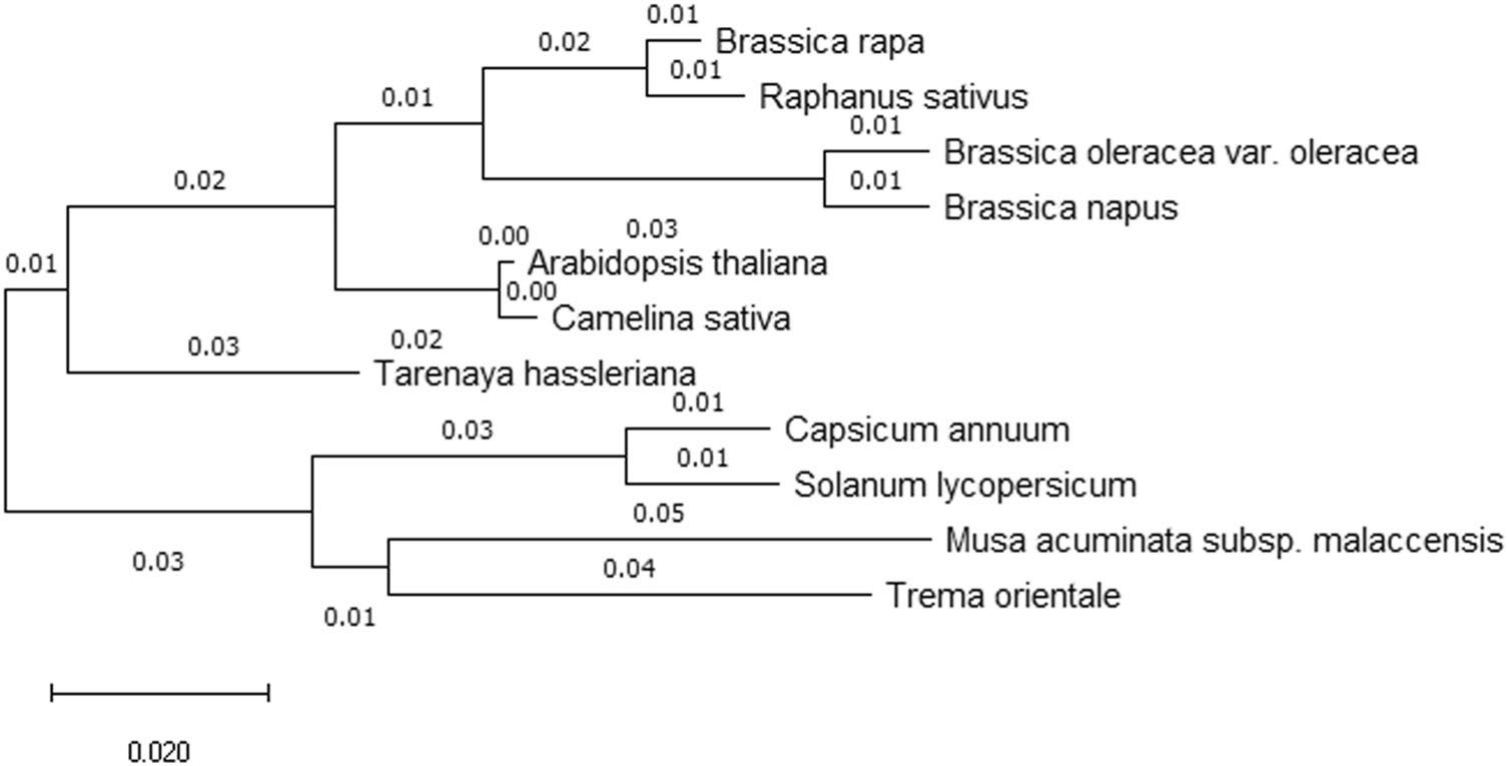
Phylogenetic tree analysis of AHA2. The tree is drawn to scale. Numbers on the tree represented branch lengths that indicate evolutionary distance. Evolutionary analyses were conducted in MEGA X 10.0.5 software (https://www.megasoftware.net).

No significant difference was observed between the enzyme activity of AHA2 in *lcm* and ‘FT’ (Table S6). The TMHMM result showed that AHA2 had eight transmembrane regions comprising amino acids at positions 242–264, 277–299, 644–666, 671–690, 710–732, 753–770, 785–807, and 814–833 (Fig. S6). The mutation site was present in the extracellular region. The SWISS-MODEL results showed that the protein tertiary structure was altered which amino acid residue of mutation site was changed, the spatial structure of AHA2 did not change as illustrated in Fig. S7. To understand the phylogenetic relationship between AHA2 and other species, a phylogenetic tree was constructed based on an NCBI BLAST search. According to the amino acid sequence, ten other homologous proteins were selected from other species as follows: *Raphanus sativus*, *Brassica oleracea* var. *oleracea*, *Brassica napus*, *Arabidopsis thaliana*, *Camelina sativa*, *Tarenaya hassleriana*, *Capsicum annuum*, *Solanum lycopersicum*, *Musa acuminata* subsp. *malaccensis*, *Trema orientale* (Fig. 7). The result indicated that AHA2 demonstrated the highest homology with *Raphanus sativus* and a homology rate was up to 98.52%.

## Discussion

In this study, the *lcm* mutant was obtained by EMS mutagenesis combined with isolated microspore culture, from the wild type ‘FT’, a DH line in Chinese cabbage. The phenotype of the *lcm* mutant was controlled by a single recessive nuclear gene, which was preliminarily mapped to chromosome A01 by BSR-Seq, and ultimately mapped to the region between markers SSRS-1 and IndelD-20. The physical distance was approximately 126.69 kb, containing twenty-three genes. The result of cloning and co-segregation verification revealed an SNP in *BraA01g007510.3C* where a C to T transition caused an amino acid codon to change from Thr (ACT) to Ile (ATT). Therefore, the *BraA01g007510.3C* was identified as the most likely candidate gene for *Bralcm.* These findings provide a solid foundation for functional analysis of *Bralcm* and promote the understanding of the molecular mechanism of leaf flattening in Chinese cabbage.

Some known genes, which are involved in the development of leaf morphology, can affect leaf flattening. For example, the *KNOX* gene was over-expressed in the cotyledons and leaves of transgenic plants with a wrinkled phenotype^25–28^. *OsAS2* in rice showed high homology with *Arabidopsis ASYMMETRIC LEAVES2* (*AS2*) which belongs to the *LBD* gene family, and the over-expression of *OsAs2* in rice caused aberrant twisted leaves with abnormal structure which lacked auricles^14^. Both RNA interference (RNAi) of *YAB3* and over-expression of *WOX3* showed a twisted leaf phenotype in rice^29^. Ren et al.^30^ found that leaf flattening is related to miRNA levels. Over-expression of *miR166* can cause rosette leaves to change from flat to downward curving, and over-expression of *miR319* can cause wavy leaf in Chinese cabbage. Mao et al.^31^ verified that over-expression of *miR319a* can silence the miR319a-targeted *Transcription factor* (*TCP*) gene and lead to wavy leaf in Chinese cabbage, which results from excess cell divisions in the leaf margins. In this study, the *BraA01g007510.3C* was the strongest candidate gene, which had a base substitution resulting in a missense mutation. *BraA01g007510.3C* encodes a homolog of AHA2, one of the key enzymes in plant growth and development, which can regulate enzyme activity, is involved in cellular transmembrane transport, and the response of plants to environmental stress^32,33^. For the first time, we discovered that *AHA2* is associated with leaf flattening in Chinese cabbage. It is important to study the function and regulatory role of *AHA2* in leaf flattening.

A total of 11 H^+^-ATPase homologous genes (*AHA1–11*) have already been identified in *A. thaliana*^34^. Further, an *Arabidopsis* homozygous loss-of-function mutant of the *AHA2* gene has been isolated and identified. The homozygous loss-of-function mutants grow normally; however, the proton secretion activity of the roots in the *aha2* mutant is decreased. Reduced membrane potential (high exogenous potassium) or pH gradient (high exogenous pH) leads to decreased growth of *aha2* mutant compared to the wild-type^35^. The present study shows that *AHA2* is associated with leaf flattening and the mutation resulted in a crinkled leaf phenotype. Previous studies showed that *AHA2* affects the growth and development of roots in *A. thaliana*, and the root phenotype of the loss-of-function mutants of *aha2* was remarkably shorter than that of the wild-type^36,37^. We observed the root phenotype at different periods as shown in Fig. S8 and expression of *BraA01g007510.3C* was analyzed by qRT-PCR. The result showed that the length of mutant root was significantly shorter than the ‘FT’, along with significantly down-regulated expression levels of *BraA01g007510.3C* compared to ‘FT’. This result is consistent with a previous study^36^ (Fig. S9). Our findings revealed that *AHA2* may affect root development in the *lcm* mutant, and a correlation may exist between root and leaf development. The electrochemical gradient generated by proton pump is the main factor affecting plant nutrient absorption, nutrient status, and root biomass^38^. The Thr^586^ mutation in extracellular region was near the Asp^588^, which is an ATP binding site. Previous study reported that the mutation near the binding site could affect the ability of binging site^39^. Therefore, we hypothesized that *AHA2* mutation may influence the ability of ATP binding, thus, affecting the development of root, thereby affecting the absorption of nutrients, eventually leading to the crinkled phenotype. Correlation of the growth of aerial and underground plant portions prompted us to analyze the expression of *BraA01g007510.3C* in leaves, thus revealing that the leaf expression levels of *BraA01g007510.3C* in the mutant were up-regulated. Thus, we hypothesize that *AHA2* may directly influence the leaf flattening and the regulatory mechanism needs to be clarified in the future. Furthermore, the relationship between *AHA2* and root and leaf development requires further investigation.

The results of the study indicated that the expression levels of *BraA01g007510.3C* were up-regulated in *lcm* mutant. SNP variations in the intron or promoter regions may lead to changes in expression levels in mutant^40^. However, sequencing results showed that the sequences of *BraA01g007510.3C* in the intron or promoter regions did not have any differences between ‘FT’ and *lcm* mutant. However, we found an SNP variation in the seventh exon of *BraA01g007510.3C*, and the expression levels were increased. *AHA2* gene was responsible for adaptation of plants to different environments. The activity and expression levels of the protein were modulated through a variety of regulatory mechanisms to adapt to the environment during the development process of plants^15^. Previous studies reported that with the changes in external environmental conditions, the levels of plant mRNA will change^40^. Mito et al.^40^ reported that *pma2* gene was homologous to *AHA2* in tomato, and the expression of *pma2* was activated when the root was exposed to adverse environmental conditions. Therefore, we speculated that when *lcm* mutant and wild-type were grown in the same environment, due to the mutation of *Bralcm* gene, the roots of the *lcm* mutant grew weaker and the ability to absorb nutrients may be weakened, resulting in less nutrients being transported to the aerial part. The aerial part may adapt to the nutrient starving conditions by improving the expression of *AHA2*.

Previous studies have shown that proton dynamics generated by H^+^-ATPase directly affect cell expansion^41^. Cellular shape is supported by the cell wall^42^. The acid growth theory was put forward to explain the cellular expansion^43–45^. Cell acidification activates expansions and other cell wall remodeling enzymes, resulting in loosening of the cell wall components and cell expansion^45–47^. However, these studies could not fully clarify the reason behind the crinkled phenotype. Therefore, SEM and paraffin sections were employed to observe the form of leaf cells. Results showed that the epidermis of leaf cells was crinkled and irregular, and the number of palisade cells was higher than that in ‘FT’. Therefore, we hypothesized that the crinkled phenotype is a result of the changes involving the epidermis and palisade cells. The leaf expression levels of *BraA01g007510.3C* in the mutant was up-regulated and resulted in the increased secretion of H^+^, and led to acidification outside the cell wall and activation of the cell wall remodeling enzymes. This resulted in a loosened cell wall, disintegration of cellulose and other components, irregular epidermis, and an increase in the number of palisade cells, ultimately, causing a crinkled phenotype. To clarify whether changes in enzyme activity were responsible for the phenotype, we measured the enzyme activity using the H^+^-ATPase ELISA kit. The result showed that the enzyme activity of the mutant and ‘FT’ was typically unchanged (Table S6). Previous study reported that the H^+^-ATPase activity is regulated by domains, such as the C-terminal domain, which played a key role in the regulation of enzyme activity through phosphorylation statua and bound effector proteins^48^. Removal of the C-terminal domain resulted in a significantly increased enzyme activity^48^. In this study, however, the mutant locus was not present in the domain. Therefore, we speculated that the unaltered enzyme activity might be due to the absence of mutations in the domains that affect enzyme activity. The reason for crinkled leaf may be due to the changes in the expression of *AHA2* gene, which affected the cell numbers without changing its enzyme activity in mutant.

In conclusion, *Bralcm* was mapped to a region of approximately 126.69 kb on chromosome A01, and *BraA01g007510.3C* was predicted to be the most likely candidate gene. *BraA01g007510.3C* encodes a homolog of *AHA2*, a vital enzyme involved in plant growth and development. For the first time, we discovered that *AHA2* could be associated with leaf flattening in Chinese cabbage. Our findings will contribute to further research on the molecular mechanism of leaf flattening in Chinese cabbage.

## Materials and methods

### Plant material

The mutant *lcm* was derived from ‘FT’, a Chinese cabbage doubled haploid (DH) line, by isolated microspore culture combined with EMS mutagenesis. The isolated microspores were treated with 0.16% EMS solution for 15 min, according to the method of Huang et al.^49^. The *lcm* mutant exhibited stable inheritance after multiple generations.

### Ploidy determination

The ploidy level of the *lcm* mutant was determined by FACSCalibur Flow Cytometer, according to the method of Huang et al.^24^. Three biological replicates were performed for each sample.

### Morphological observation of *lcm* mutant

Five consistent *lcm* mutant and wild-type ‘FT’ plants were selected for investigation. When the third true leaf appeared, the leaf length, leaf width, and plant width were measured every 3 days for a total of 22 days. Three *lcm* mutants and three ‘FT’ plants were randomly selected every 6 days to measure dry weight and fresh weight for a total of 31 days. At the heading stage, five plants were selected to measure the length, width and weight of the leafy head between *lcm* mutant and ‘FT’.

### Observation of leaf cell

To observe the shape of a leaf cell, the third true leaves with the same part from *lcm* mutant and ‘FT’ were examined by SEM as previously described by Lin et al.^50^. A fresh leaf of *lcm* mutant and ‘FT’ with the same part was fixed in FAA (50% ethanol, 5% glacial acetic, 10% formalin) for 24 h at room temperature (25 °C), dehydrated with different concentrations of alcohol (50–100%) and then permeated in xylene. Finally, the sample was embedded in paraffin. The paraffin section was processed by a microtome (LeicaRM2016, Germany); after staining, the leaf was observed using an optical microscope (Nikon ECLIPSE 80i, Japan).

### Genetic analysis of *lcm* mutant

To confirm the genetic analysis of the leaf crinkled phenotype, the *lcm* mutant was crossed with ‘FT’ to construct F_1_, F_2,_ and BC_1_ generations. The separation ratio of the F_2_ and BC_1_ generations was determined using a chi-squared (χ^2^) test.

### BSR-Seq analysis

The *lcm* mutant was crossed with ‘701’, a cabbage Sprouts DH line, whose genetic background was very different from the *lcm* mutant, to obtain an F_2_ segregation population for BSR-Seq and fine mapping. Fifty mutant and fifty normal phenotypes were selected from the F_2_ population, and sampled at the same part and stage to construct two mixed pools. Total RNA was extracted using RNApure Total RNA Kit (Aidlab, Beijing, China).

Approximately 5 GB of raw reads were obtained from the transcriptome sequencing on an Illumina HiSeq platform GENEWIZ (Suzhou, China). Clean reads were obtained by filtering low quality data and removing contamination and adaptors. The clean reads were aligned to the reference genome (https://brassicadb.org/brad/datasets/pub/Genomes/Brassica_rapa/V3.0/) using HISAT software (v2.0.14) with the default parameters^51^. By comparing the results of each sample with the reference genome, mpileup processing was carried out with samtools (v0.1.18) software to obtain the single nucleotide variants (SNVs)^52^. ANNOVAR (v2013.02.11) was used for SNV annotation. ED was calculated to estimate the genetic distance between the SNVs and the target trait: the larger the ED value, the closer the SNV is to the target trait^53^. To eliminate background noise, the ED value of each SNV was processed to the power of 5 (ED^5^). All ED^5^ values were ranked, the SNV corresponding to the top 1% of ED^5^ values were screened, and the specific chromosome regions related to the target trait were further located according to the distribution of these SNV.

### Development of molecular markers

The SSR molecular markers were designed according to the result of BSR-Seq. The polymorphic markers were screened between *lcm* and ‘701’, after linkage markers were identified, new SSR and indel markers were designed using Primer Premier 5.0 software for fine mapping. The genetic linkage map was constructed with Map-Makerv3.0^54^ and genetic map distances (cM) of recombination frequencies were transformed using Kosambi’s mapping function^55^.

### Candidate gene analysis

Primers were designed to amplify the candidate gene in *lcm* and ‘FT’. The amplified products were purified using a Gel Extraction Kit (Omega, USA). Then the purified products were inserted into the pGEM-T Easy Vector (Promega, USA) and transformed into Top10 competent cell (CWBIO, Beijing, China). The sequences were compared using DNAMAN software.

### Expression analysis by qRT-PCR

To analyze the expression pattern of the candidate gene, total RNA was extracted from roots and leaves at different periods, and from stems, flowers, and flower buds in mutant *lcm* and wild-type ‘FT’. First-strand cDNA was synthesized using the FastQuant RT Super Mix (TIANGEN, Beijing, China). The cDNA was used as a template for qRT-PCR with the SYBR Green PCR Master Mix (TaKaRa, Dalian, China). The qRT-PCR primers are shown in Table S5. The reaction volume and PCR program were as described by Huang et al.^49^. The melting curves were established to detect primer dimers. The relative gene expression data were calculated using the 2^−ΔΔCt^ method^56^. All reactions were performed using three biological replicates and the data were analyzed using the Bio-Rad IQ5 Manager software.

### Enzyme activity assay

The plant H^+^-ATPase level was measured by an ELISA Kit (Meimian Biotech Co, Ltd, Yancheng, China). Leaves and roots (1 g) were cut into pieces and added to 9 ml PBS (pH 7.2–7.4), followed by centrifugation and collection of the supernatant. The experimental process was conducted according to the manufacturer instructions of H^+^-ATPase kits^57,58^.

### Bioinformatics analysis of AHA2

The transmembrane domain of AHA2 was predicted by TMHMM Server v.2.0 online software (https://www.cbs.dtu.dk/services/TMHMM-2.0/). The protein tertiary structure of AHA2 was predicted by SWISS-MODEL software (https://swissmodel.expasy.org/). The phylogenetic tree of AHA2 was constructed by MEGA X 10.0.5 software with Clustal W and neighbor-joining based on 1,000 bootstrap replications.

## Supplementary information

Supplementary Legends.

Supplementary Information
