# Supplementary material for: Fine mapping of a leaf flattening gene Bralcm through BSR-Seq in Chinese cabbage (Brassica rapa L. ssp. pekinensis)
Source: Sci Rep. 2020 Aug 18;10:13924. doi: 10.1038/s41598-020-70975-2 (PMC7435182; doi:10.1038/s41598-020-70975-2)
Supplement: Supplementary file 1 — Supplementary Legends. [file 41598_2020_70975_MOESM1_ESM.doc]

**Supplementary Materials**

**Fig. S1** Ploidy identification by flow cytometer. (**a**) ‘FT’, (**b**) *lcm*.

**Fig. S2** Growth curve at the seedling stage. (**a**) leaf length, (**b**) leaf width, (**c**) plant width, (**d**) fresh weight, (**e**) dry weight.

**Fig. S3** ED^5^ distribution of filtered SNPs on chromosomes by BSR-Seq. Note: X-axis represents the number of different SNV sites in each chromosome of *Brassica rapa* and Y-axis represents the ED^5^ values of filtered SNPs. The horizontal line is the correlation threshold of the top 1%.

**Fig. S4** Screening of molecular markers tightly linked to *Bralcm*. (**a**) Screening of polymorphic SSR markers between the two parents. (**b**) Recombinants with SSRHG-1 in the mapping population. (**c**) Recombinants with SSRG-9 in the mapping population. P_1_: mutant *lcm.* P_2_: DH line ‘701’. Asterisks indicate recombinants.

**Fig. S5** Alignment of the nucleotide sequences of *BraA01g007510.3C* from mutant *lcm* and wild-type ‘FT’.

**Fig. S6** The transmembrane domain predicted of AHA2.

**Fig. S7** Protein tertiary structure analysis of AHA2.

**Fig. S8** Comparison of root length in *lcm* and ‘FT’ at different periods.

**Fig. S9** Expression analysis of *BraA01g007510.3C* in root with different periods by qRT-PCR (the root of cotyledon, the root of first true leaf, the root of third true leaf, the root of sixth true leaf, the root of rosette leaf, the root of head leaf). Note: Asterisks indicate significant difference between the *lcm* and ‘FT’ (t test, P < 0.05).

**Table S1** Identification of agronomic characters at the heading stage. (XLSX)

**Table S2** Summary of clean Illumina RNA-Seq reads from the two samples. (XLSX)

**Table S3** Target region of *Bralcm* using BSR-Seq. (XLSX)

**Table S4** Primer sequences of SSR and indel markers. (XLSX)

**Table S5** Candidate genes within the located region. (XLSX)

**Table S6** Enzyme activity of *lcm* and ‘FT’. (XLSL)
