# Supplementary figures and images for: Fine mapping of a leaf flattening gene Bralcm through BSR-Seq in Chinese cabbage (Brassica rapa L. ssp. pekinensis)
Source: Sci Rep. 2020 Aug 18;10:13924. doi: 10.1038/s41598-020-70975-2 (PMC7435182; doi:10.1038/s41598-020-70975-2)

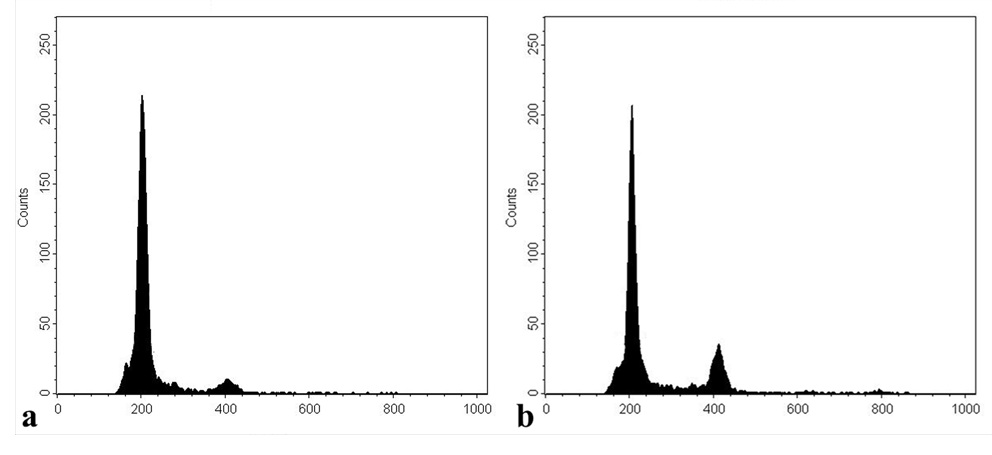

Supplement: Supplementary file 2 — Supplementary Information [file 41598_2020_70975_MOESM2_ESM.zip › Supplementary Figure S1.tif]

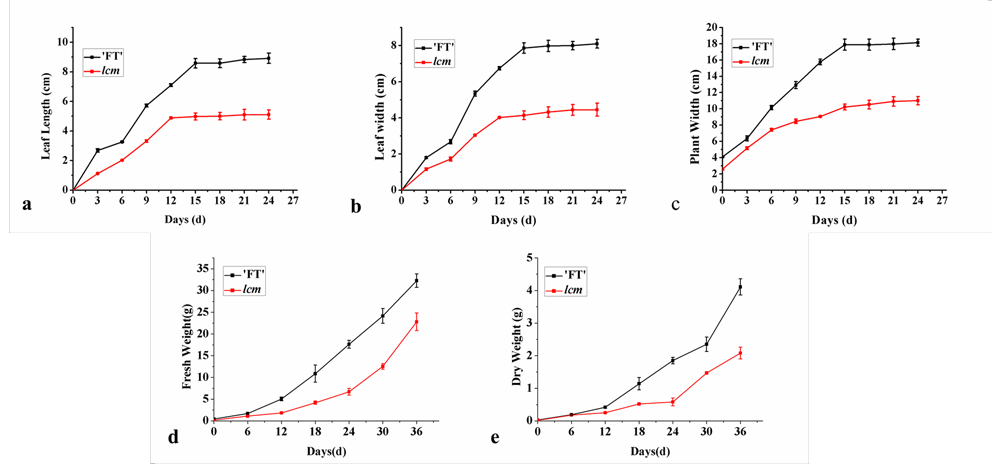

Supplement: Supplementary file 2 — Supplementary Information [file 41598_2020_70975_MOESM2_ESM.zip › Supplementary Figure S2.tif]

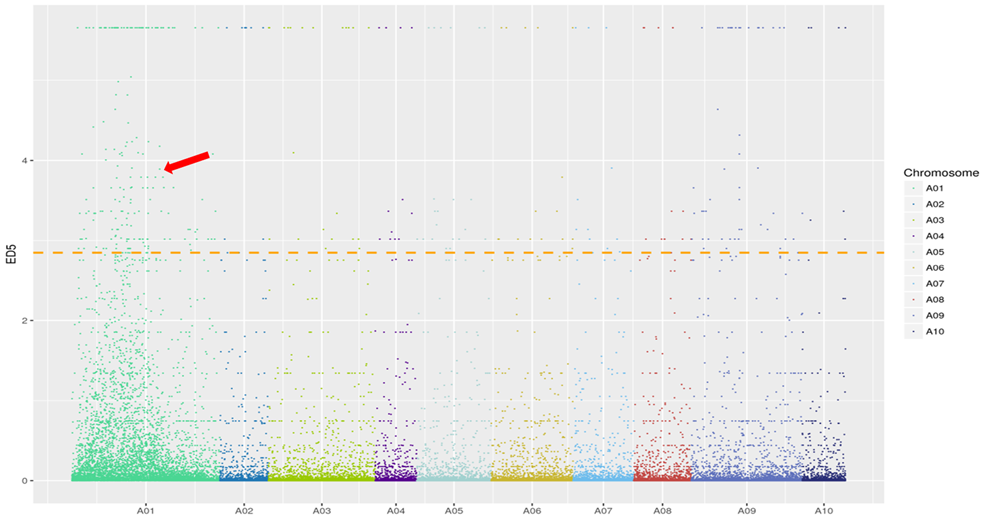

Supplement: Supplementary file 2 — Supplementary Information [file 41598_2020_70975_MOESM2_ESM.zip › Supplementary Figure S3.tif]

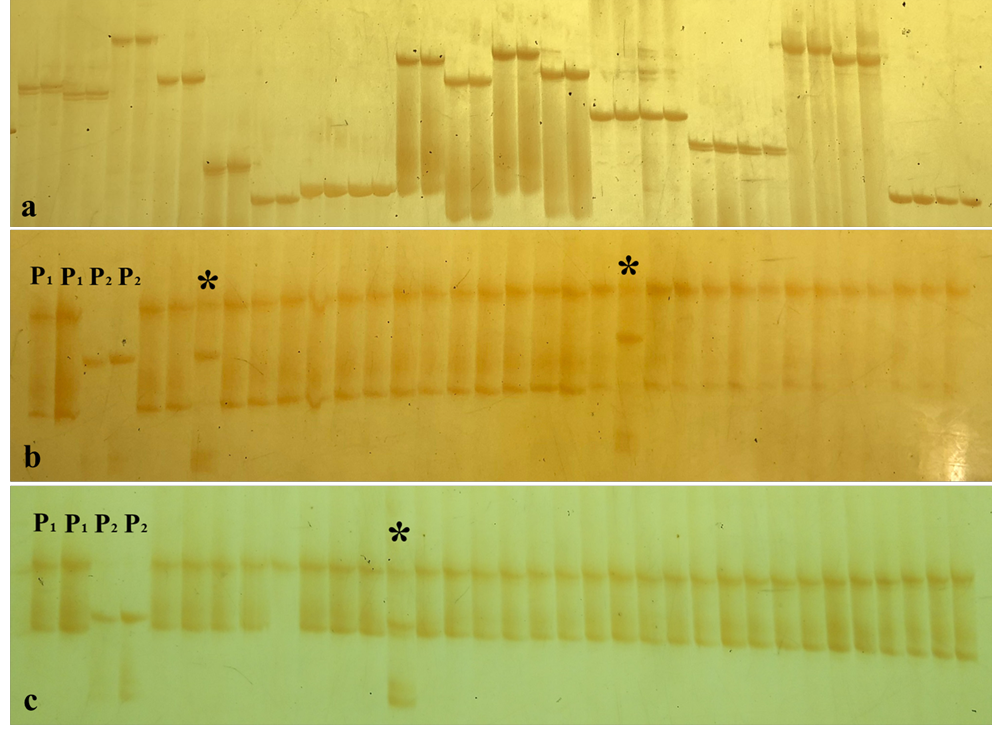

Supplement: Supplementary file 2 — Supplementary Information [file 41598_2020_70975_MOESM2_ESM.zip › Supplementary Figure S4.tif]

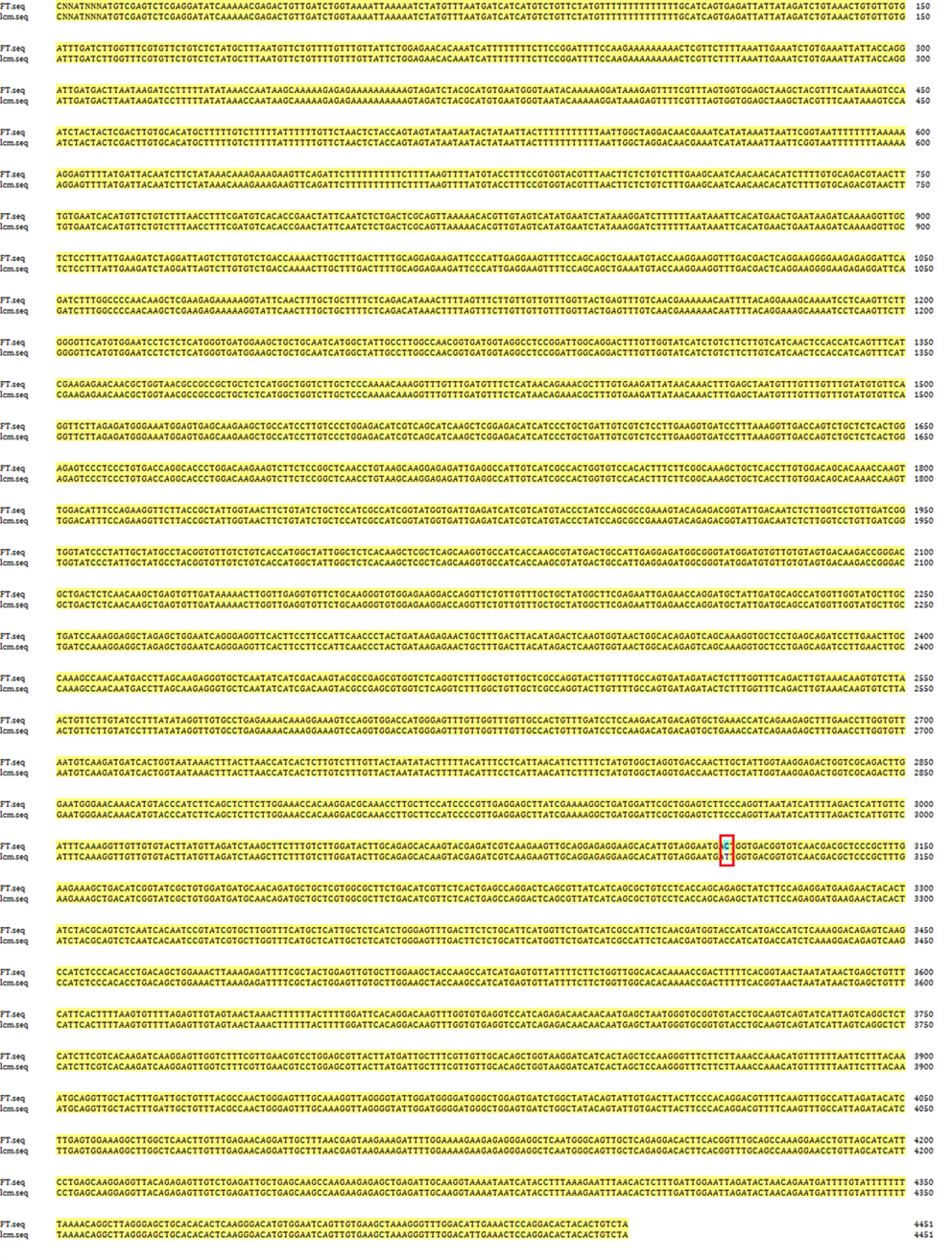

Supplement: Supplementary file 2 — Supplementary Information [file 41598_2020_70975_MOESM2_ESM.zip › Supplementary Figure S5.tif]

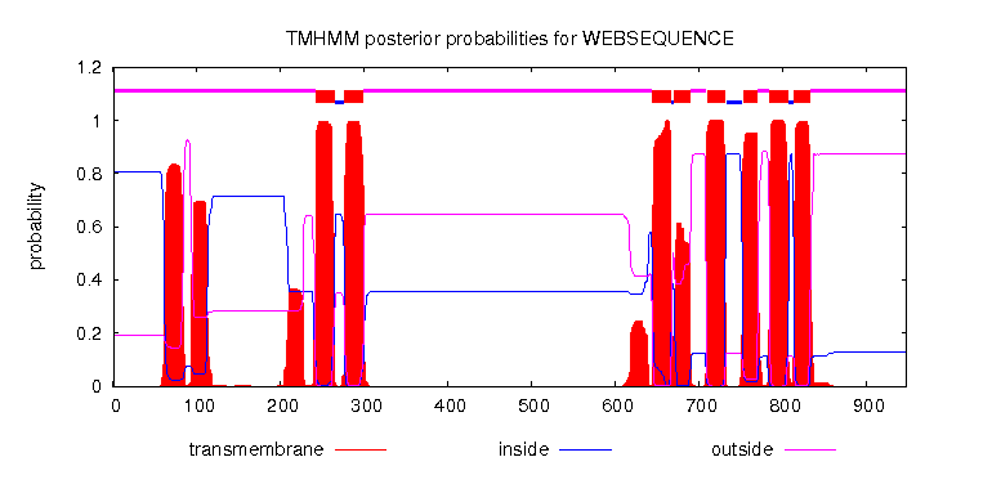

Supplement: Supplementary file 2 — Supplementary Information [file 41598_2020_70975_MOESM2_ESM.zip › Supplementary Figure S6.tif]

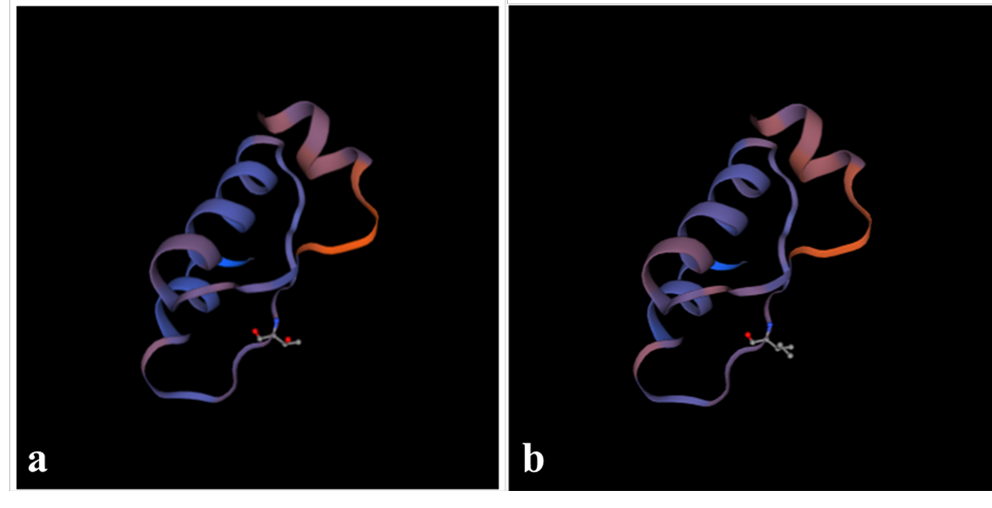

Supplement: Supplementary file 2 — Supplementary Information [file 41598_2020_70975_MOESM2_ESM.zip › Supplementary Figure S7.tif]

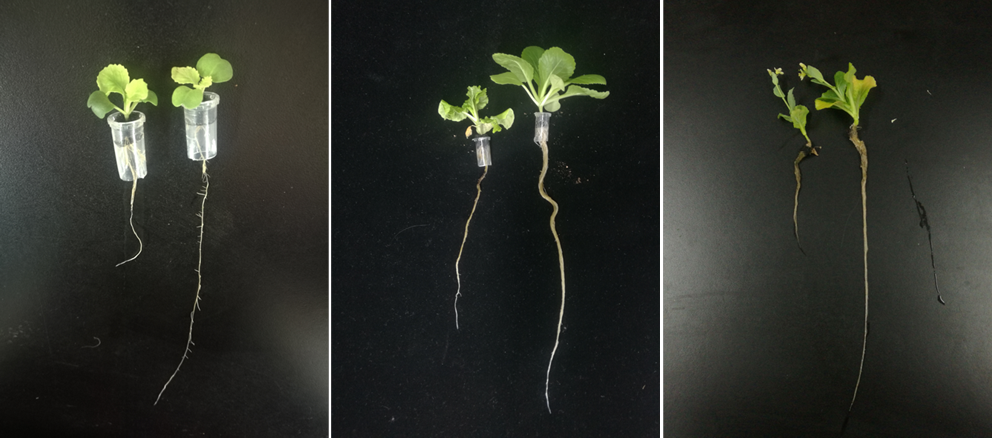

Supplement: Supplementary file 2 — Supplementary Information [file 41598_2020_70975_MOESM2_ESM.zip › Supplementary Figure S8.tif]

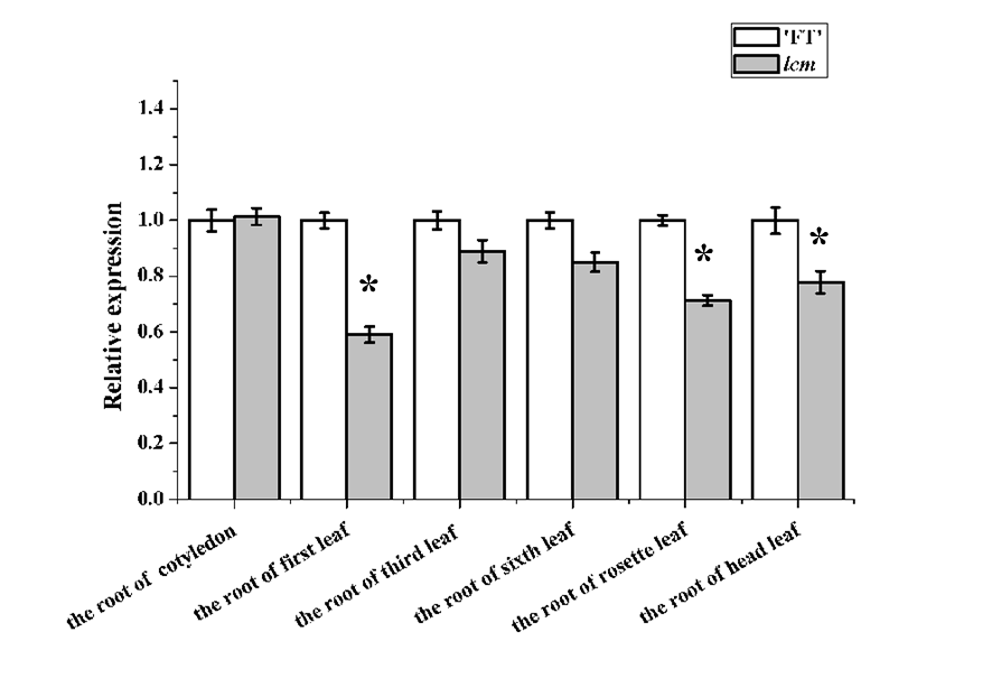

Supplement: Supplementary file 2 — Supplementary Information [file 41598_2020_70975_MOESM2_ESM.zip › Supplementary Figure S9.tif]
